# The complete mitochondrial genome of sugarcane (*Saccharum* spp.) variety FN15

**DOI:** 10.1080/23802359.2020.1768926

**Published:** 2020-05-27

**Authors:** Xiaolan Liu, Ze Yin, Ying Liu, Zhu Li, Mingli Yan, Youxiong Que, Liping Xu, Dinggang Zhou

**Affiliations:** a Hunan Key Laboratory of Economic Crops Genetic Improvement and Integrated Utilization, School of Life Science, Hunan University of Science and Technology, Xiangtan, China; b Key Laboratory of Sugarcane Biology and Genetic Breeding, Ministry of Agriculture/National Engineering Research Center for Sugarcane, Ministry of Science and Technology, Fujian Agriculture and Forestry University, Fuzhou, China; c Key Laboratory of Ecological Remediation and Safe Utilization of Heavy Metal-polluted Soils, College of Hunan Province, Xiangtan, China

**Keywords:** sugarcane, *Saccharum* spp, mitogenome, phylogenetic analysis

## Abstract

The complete mitogenome of *Saccharum* spp. hybrid FN15 was successfully sequenced. It contains two distinct circular chromosomes, Chromosome 1 and Chromosome 2. The former is 301,533 bp in length with the GC content of 43.90%, and 7.12% of genome (21,468 nucleotides) are coding DNA while 92.88% of genome (280,065 nucleotides) are intergenic region. The latter is 144,744 bp in length with the GC content of 43.57%, and 8.20% of genome (11,865 nucleotides) are coding DNA and 91.80% of genome (132,879 nucleotides) are intergenic region. Besides, Chromosome 1 contains 22 protein-coding genes (four *atp* genes, three *ccm* genes, three *cox* genes, one *mat* gene, one *mtt* gene, six *nad* genes and four *rps* genes), and 21 non-coding genes (15 tRNA and six rRNAs), whereas in Chromosome 2, there are 13 protein-coding genes (two *atp* genes, one *ccm* gene, one *cob* gene, one *cox* gene, one *rpl* gene, four *nad* genes and three *rps* genes) and five tRNA genes. Maximum Likelihood phylogenetic analysis demonstrated that FN15 is close with *S.* spp. hybrid ROC22, *S. officinarum* Khon Kaen 3 and *S. bicolor* species. This complete mitochondrial genome will provide essential DNA molecular data for further phylogenetic and evolutionary analysis for *Saccharum*.

Sugarcane (*Saccharum* spp.), which belongs to the family of Gramineae, accounts for ∼80% of sugar production and ∼60% of bioethanol production worldwide and therefore is the most important sugar and bioenergy crop (Garsmeur et al. 2018; Zhou et al., 2020). Modern commercial sugarcane varieties are all complex interspecies hybrids (Garsmeur et al. 2018; Zhou et al. 2018). FN15, a *Saccharum* spp. hybrid from CP72-1210 × YN73-204, is a national identified commercial variety and is approved to be planted in main sugarcane production provinces since 2005 in China (Zhang and Govindaraju 2018) and serves as one of the important crossing parents. The characterization of the complete mitogenome of sugarcane variety FN15 and its phylogenetic relationship within Gramineae were described in the present study.

The sugarcane variety FN15 was sampled from Fujian Agriculture and Forestry University, Fuzhou, Fujian Province (geographic coordinates: 26°9′8″N, 119°24′24″E), China. The specimen of sugarcane FN15 was stored in the Key Laboratory of Sugarcane Biology and Genetic Breeding, Fujian Agriculture and Forestry University with store number FN15- FJ2016001. An improved protocol, which reported by Chen et al. (2011), was firstly adopted to extract and purify the mitochondrial DNA (mtDNA) from fresh yellowing seedlings. Then, the complete mitochondrial genome was sequenced using Illumina Hiseq XTen and PacBio Sequel platform, and assembled by SPAdes v3.10.1 (Antipov et al. 2016). Thereafter, the genome sequences were annotated using GeSeq (Tillich et al. 2017). The complete mitochondrial genome sequence has been submitted to GenBank with the accession numbers: MT411890 (Chromosome 1) and MT411891 (Chromosome 2).

The complete mitogenome of sugarcane variety FN15 includes two distinct annular chromosomes, termed as Chromosome 1 and Chromosome 2. The Chromosome 1 is 301,533 bp in length with the GC content of 43.90%, and 7.12% of genome (21,468 nucleotides) are coding DNA while 92.88% of genome (280,065 nucleotides) are intergenic region. The latter is 144,744 bp in length with the GC content of 43.57%, and 8.20% of genome (11,865 nucleotides) are coding DNA and 91.80% of genome (132,879 nucleotides) are intergenic region. Interestingly, Chromosome 1 contains 22 PCGs (protein-coding genes, four *atp* genes, three *ccm* genes, three *cox* genes, one *mat* gene, one *mtt* gene, six *nad* genes and four *rps* genes), and 15 tRNAs and six rRNAs non-coding genes, whereas Chromosome 2 includes five tRNA genes and 13 PCGs (four *nad* genes, three *rps* genes, two *atp* genes, one *ccm* gene, one *cob* gene, one *cox* gene, and one *rpl* gene). Regarding the initiation and stop codon, all these PCGs in Chromosome 1 use the initiation codon ATG except for *nad1* and *matR*, which start with ACG and ATA, respectively, and most of them terminate with TGA, TAG, or TAA, whereas *nad2* and *nad5* stop with CGG and GTA, respectively. However, in Chromosome 2, all these PCGs begin with ATG except for *nad2* and *nad5*, which start with TTG and CCA, respectively, and *ccmFc*, *rps12*, *cox3*, and *nad4* terminate with TGA, whereas *atp9*, *rps3*, and *cob* terminate with TAG, and the other genes (*atp6*, *nad3*, *rps4*, *rpl16*, *nad2*, and *nad5*) terminate with TAA.

To explore the phylogenetic position of *Saccharum* spp. hybrid FN15, a phylogenetic tree by Maximum-Likelihood with 1000 bootstrap replications was constructed based on the whole mitochondrial genomes of 10 species using PhyML v3.0 (http://www.atgc-montpellier.fr/phyml/). GenBank accession numbers for each plant species are as follows: *Saccharum* spp. hybrid ROC22 (SRR11358604), *Oryza sativa* (NC_011033.1), *Zea mays* (NC_007982.1), *Sorghum bicolor* (NC_008360.1), *Triticum aestivum* (NC_037304.1), *Hordeum vulgare* (IBSC_v2.dna. Mt: 1:525599:1 REF), *Saccharum officinarum* Khon Kaen 3 (LC107874.1 and LC107875.1), *Arabidopisis thaliana* (NC_037304.1), and *Brassica napus* (NC_008285.1), with the last two species used as outgroups. The result indicated that the phylogenetic relationship of *Saccharum* spp. hybrid FN15 is very close to *S.* spp. hybrid ROC22 (99%), *S. officinarum* Khon Kaen 3 (99%), and *S. bicolor* (98%), which all belong to the species of family Gramineae (Figure 1). The complete mitochondrial genome sequence will provide essential and important DNA molecular data for phylogenetic and evolutionary analysis for *Saccharum*. Significant DNA molecular data offered by the complete mitochondrial genome sequence herein will contribute to further phylogenetic and evolutionary analysis of *Saccharum*.

**Figure 1.**
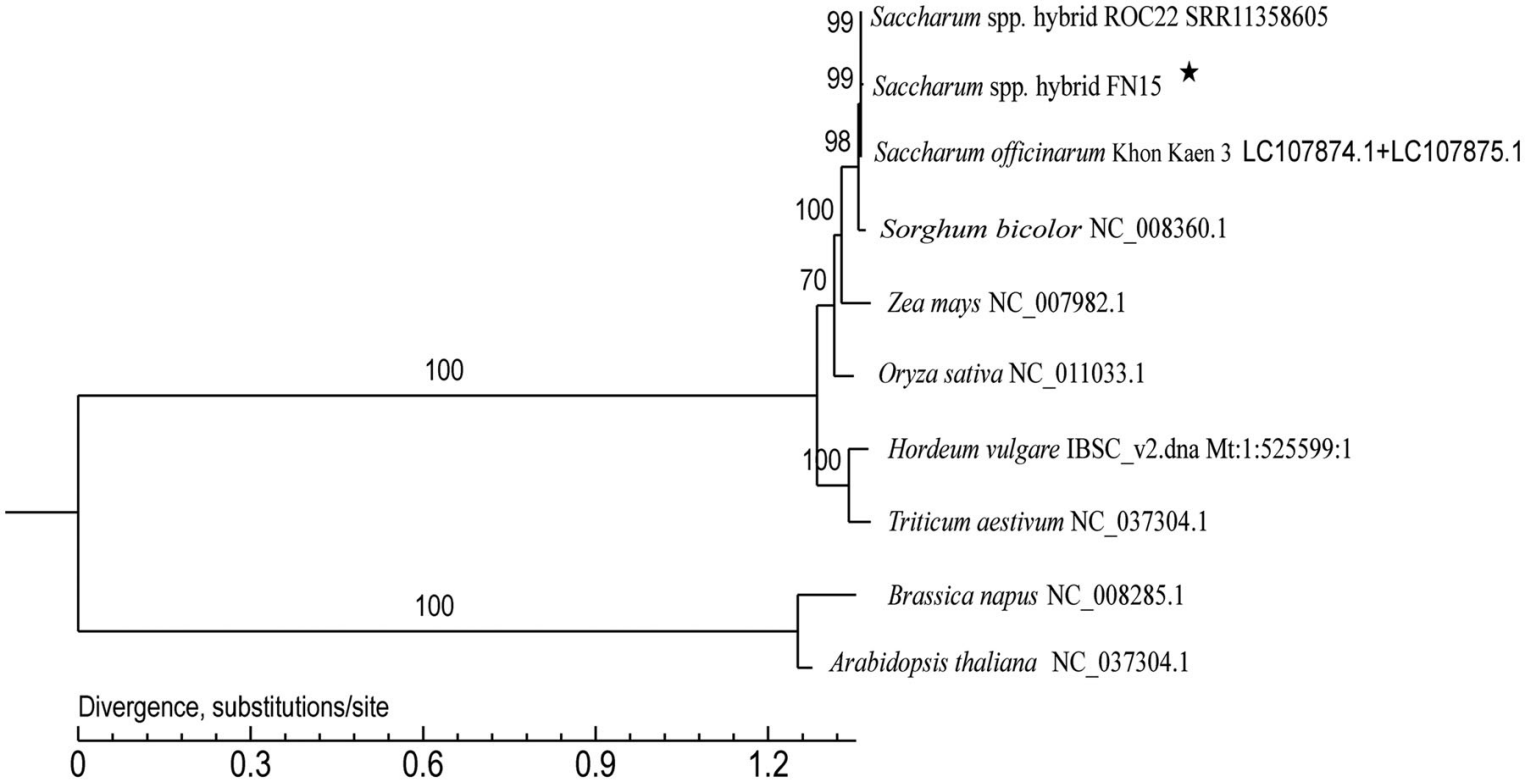
A maximum-likelihood phylogenetic tree constructed based on the comparison of mitochondrial genome sequences of ten species. GenBank accession numbers are listed after the species name. The numbers at the nodes are bootstrap percent probability values based on 1000 replications. The genome sequence in this study is labeled with an asterisk.

## Data Availability

The data that support the findings of this study are available. The mitochondrial genome sequences of analyzed species (*Saccharum* spp. hybrid FN15, *O. sativa*, *Z. mays*, *S. bicolor*, *T. aestivum*, *S. officinarum* Khon Kaen 3, *A. thaliana*, and *B. napus*) were from the GenBank databases (https://www.ncbi.nlm.nih.gov/), *Saccharum* spp. hybrid ROC22 mitchondrial genome is available in NCBI SRA with the accession number of SRR11358605 (https://dataview.ncbi.nlm.nih.gov/objects?linked_to_id=SRR11358605&archive=biosample), and the mitogenome of *H. vulgare* is available in ensembl (http://asia.ensembl.org/index.html).
